# Moral distress among nurses during COVID‐19 pandemic: Challenges and coping strategies

**DOI:** 10.1002/nop2.1248

**Published:** 2022-05-23

**Authors:** Mohammad Javad Ghazanfari, Shaqayeq Esmaeili, Amir Emami Zeydi, Samad Karkhah

**Affiliations:** ^1^ Department of Medical‐Surgical Nursing, School of Nursing and Midwifery Kashan University of Medical Sciences Kashan Iran; ^2^ Imam Khomeini Hospital Mazandaran University of Medical Sciences Sari Iran; ^3^ Department of Medical‐Surgical Nursing, Nasibeh School of Nursing and Midwifery Mazandaran University of Medical Sciences Sari Iran; ^4^ Department of Medical‐Surgical Nursing, School of Nursing and Midwifery Guilan University of Medical Sciences Rasht Iran; ^5^ Burn and Regenerative Medicine Research Center Guilan University of Medical Sciences Rasht Iran; ^6^ Quchan School of Nursing Mashhad University of Medical Sciences Mashhad Iran

**Keywords:** COVID‐19, moral distress, nurses, nursing

To the Editor,

The COVID‐19 pandemic changed the world’s image due to various unpredictable effects on health, living tradition, economy, and politics. Meanwhile, healthcare providers have faced multidimensional challenges in performing their professional duties and responsibilities during this pandemic. In particular, nurses as front‐line workforce in COVID‐19 patient care have been challenged due to a lack of pandemic preparedness, and scarce clinical resources and equipment. On the other hand, nurses endured high workload pressure due to various roles in hospital management activities such as resource mobilization during the pandemic. Tolerance of this high workload can confront nurses with numerous ethical challenges and conflicts, which, if persisted, can lead to moral distress in them (Gebreheat & Teame, 2021).

Moral distress is a negative feeling and psychological disequilibrium when a nurse makes a moral decision but feels that the decision cannot be followed due to institutional constraints despite knowing the correct moral action. Many of the challenges during the COVID‐19 pandemic led to ethical conflicts and moral distress in nurses (Cacchione, 2020). Most of these challenges are related to nurses and their relationship with patients. At the pandemic’s beginning, information deficiencies led nurses to worry about keeping themselves and their families healthy. On the other hand, the lack of personal protective equipment had exacerbated nurses’ concerns. Also, the lack of workforce and frequent shifts and overtime had put more pressure on nurses to provide care for COVID‐19 patients (Morley et al., 2020). Some of the challenges have been related to the early pandemics and have diminished over time. For example, challenges were somewhat diminished, such as shortages in personal protective equipment, intensive care unit beds, and ventilators after partial government funding. However, new challenges arose regarding the distribution and inequality of resources. It has been reported that this discrimination against nurses has led to a loss of motivation for nurses to provide quality care due to the greater dominance of physicians in receiving equipment. On the other hand, although the organization of nursing managers in terms of workforce improved due to the initial experiences of the pandemic, however, in different pandemic peaks, challenges such as high workload and shortage of workforce in nurses reappeared. On the other hand, a relative knowledge of the nature of the disease, the route of transmission, the prognosis, and the production of vaccines led to a reduction in ambiguity and fear of infecting oneself and one’s family, and, consequently, a reduction in the ethical challenges in them. However, this challenge remains until global awareness of the disease and effective vaccination against COVID‐19 is established. Due to political, economic, and educational constraints, the current challenge is discrimination in access to vaccines and hesitation in accepting COVID‐19 vaccination. Producing countries provided only vaccines to countries with good financial capacity. Therefore, access to vaccines at the global population level will be challenging (Forni & Mantovani, 2021). This discrimination in access to vaccines and hesitation in accepting COVID‐19 vaccination in the general population causes moral distress among nurses when they encounter critically ill patients whose infection and mortality could have been prevented with timely access to vaccines and accurate information about vaccines. These challenges lead to moral distress among nurses, which can negatively affect nurses, such as imposing physical and psychological stress (Forni & Mantovani, 2021).

Meanwhile, nurses must implement coping strategies to prevent and reduce the negative effects of moral distress. These strategies can have positive effects on nurses' physical and mental health. Watching movies, social communication with family and friends, walking, maintaining a sense of humour, yoga, meditation, listening to music, adequate nutrition, enough sleep, talking about their feelings with peers, and forming a moral support group are some potentially helpful coping strategies to prevent or reduce the negative effects of moral distress among nurses (Godshall, 2021). However, to the best of our knowledge, there is no research to evaluate and prove the efficacy of such potential interventions among nurses to prevent and reduce the negative effects of moral distress during the COVID‐19 pandemic.

Overall, it is essential to pay attention to the health of nurses as the leading workforce to deal with COVID‐19. Nursing managers and policymakers should pay special attention to using coping strategies to prevent or reduce the negative effects of moral distress among nurses. Due to the persistence of the COVID‐19 pandemic, researchers can provide appropriate interventions to reduce the pressure on nurses in this pandemic so that it can be used by nursing managers and policymakers. In fact, the experience gained from this pandemic can be used not only now but also in future pandemics.

## CONFLICTS OF INTEREST

The author(s) declared no potential conflicts of interest with respect to the research, authorship, and/or publication of this article.

## Data Availability

The datasets used and/or analysed during the present study are available from the corresponding author on reasonable request.
